# MRI radiomics independent of clinical baseline characteristics and neoadjuvant treatment modalities predicts response to neoadjuvant therapy in rectal cancer

**DOI:** 10.1038/s41416-022-01786-7

**Published:** 2022-04-02

**Authors:** Maxiaowei Song, Shuai Li, Hongzhi Wang, Ke Hu, Fengwei Wang, Huajing Teng, Zhi Wang, Jin Liu, Angela Y. Jia, Yong Cai, Yongheng Li, Xianggao Zhu, Jianhao Geng, Yangzi Zhang, XiangBo Wan, Weihu Wang

**Affiliations:** 1 Key Laboratory of Carcinogenesis and Translational Research (Ministry of Education/Beijing), Department of Radiation Oncology, Peking University Cancer Hospital and Institute, Beijing, China; 2 Department of Radiation Oncology, Peking Union Medical College Hospital, Chinese Academy of Medical Sciences & Peking Union Medical College, Beijing, China; 3 Department of Oncology, Tianjin Union Medical Center, Tianjin, China; 4 Blot Info & Tech (Beijing) Co. Ltd, Beijing, China; 5 Department of Radiation Oncology and Molecular Radiation Sciences, Johns Hopkins University School of Medicine, Baltimore, MD USA; 6 Department of Radiation Oncology, Department of Medical Engineering, Guangdong Institute of Gastroenterology, The Sixth Affiliated Hospital of Sun Yat-sen University, Guangzhou, China

**Keywords:** Rectal cancer, Surgical oncology, Translational research, Cancer models, Cancer imaging

## Abstract

**Background:**

To analyse the performance of multicentre pre-treatment MRI-based radiomics (MBR) signatures combined with clinical baseline characteristics and neoadjuvant treatment modalities to predict complete response to neoadjuvant (chemo)radiotherapy in locally advanced rectal cancer (LARC).

**Methods:**

Baseline MRI and clinical characteristics with neoadjuvant treatment modalities at four centres were collected. Decision tree, support vector machine and five-fold cross-validation were applied for two non-imaging and three radiomics-based models’ development and validation.

**Results:**

We finally included 674 patients. Pre-treatment CEA, T stage, and histologic grade were selected to generate two non-imaging models: C model (clinical baseline characteristics alone) and CT model (clinical baseline characteristics combining neoadjuvant treatment modalities). The prediction performance of both non-imaging models were poor. The MBR signatures comprising 30 selected radiomics features, the MBR signatures combining clinical baseline characteristics (CMBR), and the CMBR incorporating neoadjuvant treatment modalities (CTMBR) all showed good discrimination with mean AUCs of 0.7835, 0.7871 and 0.7916 in validation sets, respectively. The three radiomics-based models had insignificant discrimination in performance.

**Conclusions:**

The performance of the radiomics-based models were superior to the non-imaging models. MBR signatures seemed to reflect LARC’s true nature more accurately than clinical parameters and helped identify patients who can undergo organ preservation strategies.

## Introduction

Rectal cancer accounts for 3.4% of all cancer-related deaths globally [1]. More than 700,000 new cases of rectal cancer are diagnosed annually worldwide, with ≥30% accounting for locally advanced rectal cancers (LARCs) [1, 2]. Neoadjuvant (chemo)radiotherapy combined with total mesorectal excision (TME) is one of the standard treatments for LARC [3, 4]. LARC is known to be a heterogeneous disease with wide variations in response to neoadjuvant (chemo)radiotherapy. Clinical individual-level surrogate response to neoadjuvant (chemo)radiotherapy is evaluated using pathological tumour regression grade (TRG) and downstaging [5, 6], and both of them can be used as treatment monitoring and prognostic parameters [7, 8]. Approximately 15–27% of patients show a pathologic complete response (pCR), which has been demonstrated to be a favourable prognostic marker [9]. For patients with clinical complete response (cCR), organ preservation strategies, such as the “wait-and-see” strategy and local excision, can achieve a similar survival rate with pCR compared to TME, thus reducing TME-related morbidity and functional complications [10, 11]. Therefore, accurately predicting tumour response in a timely and non-invasive manner before administering neoadjuvant (chemo)radiotherapy is urgently needed in individualised medical treatment for LARC, especially for identifying patients who can benefit from organ preservation strategies.

Previous studies have developed a non-imaging clinical risk model to predict complete response (CR) to neoadjuvant (chemo)radiotherapy with the area under the curve (AUC) between 0.609 and 0.706 [12–14]. Modern imaging techniques, such as conventional magnetic resonance imaging (MRI) and functional MRI, have facilitated the recognition of different responses [15]. However, accurate detection of CR using visual judgment imaging techniques remains challenging in clinical practice [15, 16]. Radiomics focuses on improvements in image analysis using an automated high-throughput extraction of large amounts of quantitative features of medical images [17]. Considering the improvement in computational capabilities, radiomics has emerged a promising tool that may serve as an imaging biomarker for tumour response in rectal cancer [18, 19]. Several studies comparing radiomics with conventional imaging showed that radiomics outperformed qualitative subjective analysis on MRI [20, 21]. However, the application of previous models is limited by the relatively small samples or a single-centre cohort and the use of post-treatment imaging features or clinical variables to assess therapeutic responses [22, 23]. Moreover, the exact region of interest (ROI) delineation of the tumour post-(chemo)radiotherapy is difficult and less reproducible than that in pre-treatment analysis, especially in diffusion-weighted imaging (DWI) [24]. More importantly, different neoadjuvant treatment modalities might change the tumour response, but the possible effect of neoadjuvant treatment modalities on response prediction is seldom considered. For a patient to receive optimal treatment with the highest success rate, developing a response prediction model that considers various factors that may affect the outcome is imperative before initiating treatment.

Because of extensive attention on the high prevalence and personalised treatment for LARC but limited knowledge on the effect of neoadjuvant treatment modalities on CR (pCR and cCR) prediction, we aimed to develop and validate a multicentred prediction model that incorporated the pre-treatment (baseline) MRI radiomics features based on T2-weighted images (T2WIs), clinical baseline characteristics, and neoadjuvant treatment modalities for stratifying patients with LARC.

## Methods

### Patient selection

A total of 735 patients with rectal cancer who underwent neoadjuvant (chemo)radiotherapy at one of four centres (Peking University Cancer Hospital, The Sixth Affiliated Hospital of Sun Yat-sen University, Peking Union Medical College Hospital, and Tianjin Union Medical Centre) between January 2012 and January 2019 were retrospectively recruited. The inclusion criteria were histologically confirmed rectal adenocarcinoma with biopsy sample, patients aged at least 18 years, clinical stage T3–T4 or any stage T and N+ tumours without distant metastasis (based on the 7th edition of the American Joint Committee on Cancer [AJCC]), pre-treatment magnetic resonance (MR) examination (including a T2-weighted [T2W] sequence), and availability of either histology after radical surgery or long-term (>2 years) follow-up in case of a “wait-and-see” program for those with cCR [25]. The reference criteria for assessment of cCR included the Sao Paulo Schema, European Society for Medical Oncology (ESMO) Schema, and Memorial Sloan Kettering Regression Schema [3, 26, 27].

The exclusion criteria were patients lost to follow-up, poor-quality images or incomplete imaging data, short-course radiotherapy with surgery within 1 week, occurrence of distant failure before surgery, previous recurrent rectal cancer, incomplete neoadjuvant (chemo)radiotherapy, double primary cancer, and history of pelvic radiation.

The final cohort included 674 patients. Clinical baseline characteristics included age, sex, pre-treatment carcinoembryonic antigen (CEA) levels, histologic grade, tumour location, and MRI-predicted T stage (MRI-T stage). This multicentre study was conducted in accordance with the Declaration of Helsinki and was approved by the Ethics Committee of the Peking University Beijing Cancer Hospital and Institute (2018KT78), and the requirement for individual informed patient consent was waived owing to the retrospective nature of the study.

### Neoadjuvant treatment

The intensity-modulated radiation therapy regimen consisted of two schedules: short-course radiotherapy (25 Gy total dose at 5 Gy per fraction) and long-course chemoradiation (22–25 fractions of 2–2.3 Gy [gross tumour volume] and 1.8–2.0 Gy [clinical target volume]) [28]. Neoadjuvant treatment modalities included short-course radiotherapy followed by neoadjuvant chemotherapy, long-course chemoradiation, neoadjuvant chemotherapy followed by long-course chemoradiation, and long-course chemoradiation followed by neoadjuvant chemotherapy. For short-course radiotherapy regimen, surgery should be delayed at 6–8 weeks. Although there were some differences among the different centres, the treatment protocol and the interval to TME followed the ESMO/National Comprehensive Cancer Network guidelines combined with patient/surgeon choice [3, 4].

### Pathologic assessment of response

Every surgical specimen underwent standardised pathologic examination by two dedicated gastrointestinal pathologists who were blinded to clinical and MRI findings. Tumours were staged according to the seventh edition of the AJCC tumour-node-metastasis (TNM) classification. TRG was evaluated using the four-tier AJCC system.

### Magnetic resonance imaging (MRI) acquisition

MRI was all performed with a 3.0-T scanner at the four centres. The imaging protocol included a T2W turbo spin-echo sequence in the axial, sagittal, and coronal planes. T2 weighted MR imaging acquisition parameters of the four centres are shown in Table 1. All patients underwent MR examination in the supine position within 2 weeks before the start of treatment, and no special bowel preparation was performed.

**Table 1:** T2 weighted MR imaging acquisition parameters of the four centres.

Parameter	Scanner	TR (ms)	TE (ms)	FOV(mm)	Flip Angle	Matrix	Slice thickness/gap (mm)	Pixel size (mm)
Centre 1	SIEMENS 3.0 T (Magnetom Skyra)	4000	107	400 × 400	150–175°	640 × 640	4/0	0.625 × 0.625
Centre 2	GE 3.0 T (Signa HDX)	5160	151	220 × 220	90°	320 × 258	3/0.3	0.688 × 0.853
Centre 3	GE 3.0 T (OPTIMA)	4300	104	100 × 100	90°	288 × 256	3/6	0.347 × 0.391
Centre 4	SIEMENS 3.0 T (Magnetom Skyra)	4000	107	400 × 400	150–175°	640 × 640	4/0	0.625 × 0.625

*TR* repetition time, *TE* echo time, *FOV* field of view.

### Tumour segmentation

**Fig. 1:**
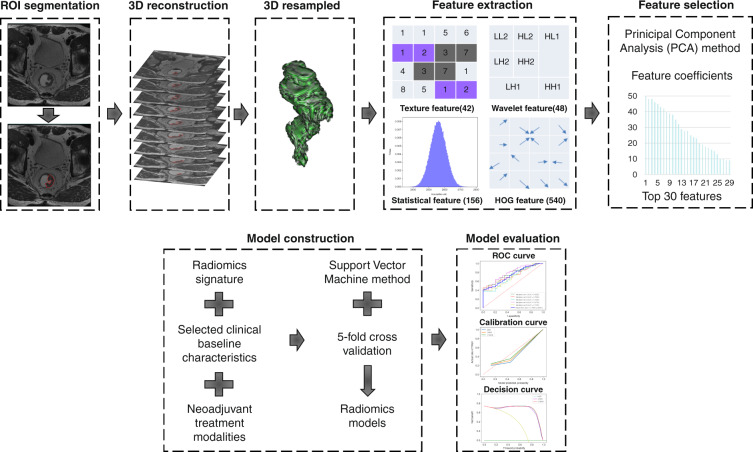
Study workflow describing the image segmentation, radiomics feature extraction, and radiomics-based prediction model building steps. The region of interest (ROI) in each transverse section manually segmented on T2 weighted magnetic resonance images (MRI). After three-dimensional reconstruction and resampling of the ROI, 786 features were extracted, and the top 30 were selected via the principal component analysis method. In addition to the MRI-based radiomics signatures, another two radiomics models based on the selected features, selected clinical baseline characteristics and neoadjuvant treatment modalities, were developed using the support vector machine method, and five-fold cross-validation was applied. The performance of all three models were evaluated using receiver-operating characteristic and calibration and decision curves.

The entire region of interests (ROIs) of rectal cancer, which covered the entire volume of the tumour, were manually drawn along the contour of the tumour on T2WI containing the surrounding chords and burrs on each slice, and the intestinal lumen was excluded (detailed ROI segmentation is shown in Fig. 1). All manual tumour segmentations were separately performed by four independent radiation oncologists with ≥5 years of experience in radiotherapy of rectal cancer. They were manually adjusted where deemed necessary by one abdominal radiologist and finally validated by one radiation oncologist with ≥20 years of experience in rectal cancer radiotherapy.

### Standardisation of MRI and extraction of radiomics features

Regarding the inhomogeneity among different MR system vendors and acquisition protocols from the four centres, we performed standardisation of MRI signal intensity to reduce the effect of the scanner. The ROIs were then extracted from the T2W sequence, and three-dimensional reconstruction was performed. Subsequently, each ROI image was resampled into 1 × 1 × 1-mm voxels, which could reduce the difference between image pixels and form a three-dimensional ROI.

Four groups of imaging features were extracted from the T2W sequence in the three-dimensional ROI: 540 histograms of oriented gradient (HOG) features, 42 texture features, 48 wavelet features, and 156 statistical features, resulting in a total of 786 features per patient. Next, invalid radiomics features (such as infinite value, null value, feature with zero variance) were removed from 786 features. Subsequently, we used the min–max normalisation method, which transformed the data into standardised intensity ranging from 0 to 1, normalising the extracted features.

### Selection of radiomics features and construction of radiomics signatures

We used the principal component analysis (PCA) method which is a multivariate statistical technique commonly applied to systematically reduce the number of dimensions needed to describe radiomics features through a decomposition process that filters features from the largest to smallest spatial scales.

The grid-search method was used to optimise the PCA parameters. When the number of retained features is 30, the optimal prediction model will be generated; hence, we chose the largest 30 eigenvalues and ignored the rest. Next, the corresponding 30 eigenvectors are used as column vectors to form the eigenvector matrix. Transformed the radiomics features of 674 patients into a new space constructed by 30 feature vectors which are the principal components after dimensionality reduction, and incorporated them into the model as effective features.

### Development and evaluation of radiomics models

Based on the results of our preliminary experiments, we finally chose the decision tree classifier to build two non-imaging prediction models. One was based on the selected clinical baseline characteristics alone termed the C model, while the other combined the selected clinical baseline characteristics and neoadjuvant treatment modalities and was termed the CT model. A support vector machine (SVM) classifier was applied to construct the MRI-based radiomics (MBR) signatures, which could identify a hyperplane that best separated CR and incomplete response, and the grid-search method was used to optimise the parameters. Similarly, a clinical individualised model was built by combining the constructed MBR signatures and the selected clinical baseline characteristics termed the CMBR model. Another individualised model based on MBR signatures, clinical baseline characteristics, and neoadjuvant treatment modalities was named the CTMBR model. Each of the selected clinical baseline characteristics and neoadjuvant treatment modalities were incorporated into these models as separate features, and their contributions to the established model were evaluated using their respective coefficients.

The 674 patients were randomly divided into five equal size subgroups because five-fold cross-validation was applied. Each subgroup was regarded as a validation set and the remaining four-fifths of the patients as the training set. This process was repeated five times with different subgroups to form five training sets and five corresponding validation sets.

Evaluation of the above models included discrimination, calibration, and clinical usefulness. Discrimination performance was quantified based on the AUC of the receiver operating characteristic (ROC) curve. The Delong test was performed to estimate whether the difference between two arbitrary ROC curves was statistically significant. Classification accuracy, positive predictive value (PPV), and negative predictive value (NPV) were also calculated to quantify the discrimination ability of the prediction models in both cohorts. Calibration curves were assessed based on the agreement between the predicted and actual CR/TRG 0 rates. Decision curve analysis was used to identify the range of threshold probabilities in which a model was of value, the magnitude of benefit, and which of the several models was optimal [29]. Study workflow describing the image segmentation, radiomics feature extraction, and radiomics-based prediction model building steps is detailed in Fig. 1.

### Statistical analyses

Sample size calculation is detailed in Supplemental Material eAppendix I. Categorical variables were compared using the *χ*^2^ or Fisher’s exact test. The independent-samples *t-*test was used to analyse continuous variables. Univariate and multivariate logistic regression analyses were performed to identify clinical baseline characteristics associated with TRG. Variables with *P* < 0.05 were selected as candidates for the model construction. A two-tailed *P*-value <0.05 was considered a statistically significant difference. The above analyses were performed using the SPSS version 23.0 (IBM, Armonk, NY, USA). The ROI, MRI signal-intensity standardisation, radiomics feature extraction, and model training were performed using the Precision Medicine Open Platform version 2.0.1 (https://www.blothealth.com). Radiomics feature selection and prediction model construction were performed using PyCharm version 2017.3.2 (https://www.jetbrains.com). Code availability: The code might be made available upon request.

## Results

### Patient characteristics

Patients were divided into the “CR” (TRG 0, *n* = 174) (including 29 patients with cCR and 145 with radical surgery) and “incomplete response” (TRG 1–3, *n* = 500) groups. Clinical baseline characteristics and neoadjuvant treatment modalities of the two groups are shown in Table 2. Pre-treatment CEA, MRI-T stage, and tumour histologic grade were significantly different between the two groups. Univariate and multivariate analyses of clinical baseline characteristics identified elevated pre-treatment CEA levels (*P* = 0.015) and MRI-T3/4 stage (*P* < 0.001) as risk factors for TRG 1–3, whereas a poorly differentiated adenocarcinoma (*P* = 0.002) was a protective factor for TRG 1–3 (Supplemental Material eTable 1).

**Table 2:** Clinical baseline characteristics and neoadjuvant treatment modalities in the TRG 0 and TRG 1–3 groups.

Characteristic	TRG 0 (*n* = 174)	TRG 1-3 (*n* = 500)	*P*
Age, mean ± SD, years	55.41 ± 11.50	56.84 ± 10.92	0.145
Sex (%)			0.250
Male	118(67.8)	362(72.4)	
Female	56(32.2)	138(27.6)	
Pre-treatment CEA(ng/ml)(%)			0.012
<5	105(60.3)	252(50.4)	
≥5	58(33.4)	223(44.6)	
NA	11(6.3)	25(5.0)	
Distance from anal verge(cm)(%)			0.658
<5	82(47.1)	217(43.4)	
5–10	90(51.7)	275(55.0)	
>10	2(1.2)	8(1.6)	
MRI-T stage(%)			<0.001
1	1(0.6)	1(0.2)	
2	15(8.6)	8(1.6)	
3	129(74.1)	377(75.4)	
4	29(16.7)	114(22.8)	
Tumour histologic grade(%)			0.012
Well differentiated adenocarcinoma	15(8.6)	58(11.6)	
Moderately differentiated adenocarcinoma	115(66.1)	303(60.6)	
Poorly differentiated adenocarcinoma	21(12.1)	30(6.0)	
Signet ring cell cancer or mucinous adenocarcinoma	1(0.6)	6(1.2)	
Uncertain differentiation type	22(12.6)	103(20.6)	
Time interval between neoadjuvant (chemo)radiotherapy and surgery, median (IQR) (weeks)	9.57(8.00–11.50)	9.57(7.86–13.14)	0.344
Neoadjuvant treatment modalities(%)			0.173
Short-Course Radiotherapy+ Neoadjuvant chemotherapy	6(3.5)	32(6.4)	
Long-Course Chemoradiation	58(33.3)	192(38.4)	
Neoadjuvant chemotherapy+ Long-Course Chemoradiation	13(7.5)	41(8.2)	
Long-Course Chemoradiation+ Neoadjuvant chemotherapy	97(55.7)	235(47.0)	

*TRG* tumour regression grade, *SD* standard deviation, *CEA* carcinoembryonic antigen, *MRI-T stage* MRI-predicted T stage, *IQR* interquartile range.

### Selection of radiomics features and construction of radiomics signatures

A total of 47 invalid radiomics features, including 4 with infinite values, 15 with null values, and 28 with variances of zero, were removed. Among the remaining 739 radiomics features, 30 features were selected for constructing the MBR signatures. Five-fold cross-validation was applied, and a total of 150 radiomics features were collected for validating the application: 77 HOG features, 8 texture features, 16 wavelet features, and 49 statistical features. The distribution of the selected radiomics features is shown in Supplemental Material (eTable 2).

### Evaluation and comparison of different prediction models

**Fig. 2:**
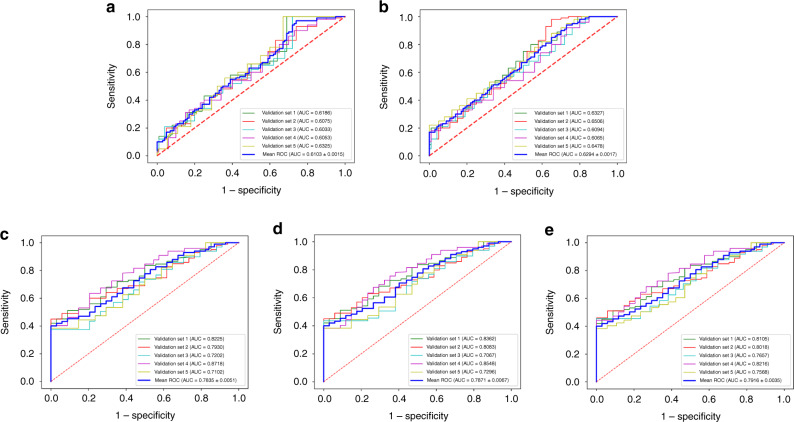
ROC curves for the C and CT and MBR and CMBR and CTMBR models. **a** ROC curve for the C model in the validation set, showing a mean AUC of 0.6103(95% CI, 0.5983–0.6286). **b** ROC curve for the CT model in the validation set, showing a mean AUC of 0.6294 (95% CI, 0.6036–0.6552). **c** ROC curve for the MBR model in the validation set, showing a mean AUC of 0.7835 (95% CI, 0.6984–0.8686). **d** ROC curve for the CMBR model in the validation set, showing a mean AUC of 0.7871 (95% CI, 0.7057–0.8686). **e** ROC curve for the CTMBR model in the validation set, showing a mean AUC of 0.7916 (95% CI, 0.7570–0.8263). Receiver-operating characteristic, ROC; clinical baseline characteristics, C; clinical baseline characteristics and neoadjuvant treatment modalities, CT; MRI-based radiomics, MBR; MBR signatures combined with clinical baseline characteristics, CMBR; MRI-based radiomics signatures combined with clinical baseline characteristics and neoadjuvant treatment modalities, CTMBR.

When considering the C model generated from the selected clinical baseline characteristics alone (pre-treatment CEA levels, MRI-T stage, and tumour histologic grade), the mean AUCs of 0.7053(95% confidence interval [CI], 0.6667–0.7440) in the training set and 0.6103(95% CI, 0.5983–0.6286) in the validation set (Fig. 2a) were obtained. Then, we combined those 3 clinical baseline characteristics and neoadjuvant treatment modalities to generate the CT model, the mean AUCs of 0.7512(95% CI, 0.6761–0.8256) in the training set and 0.6294 (95% CI, 0.6036–0.6552) in the validation set (Fig. 2b) were obtained.

**Fig. 3:**
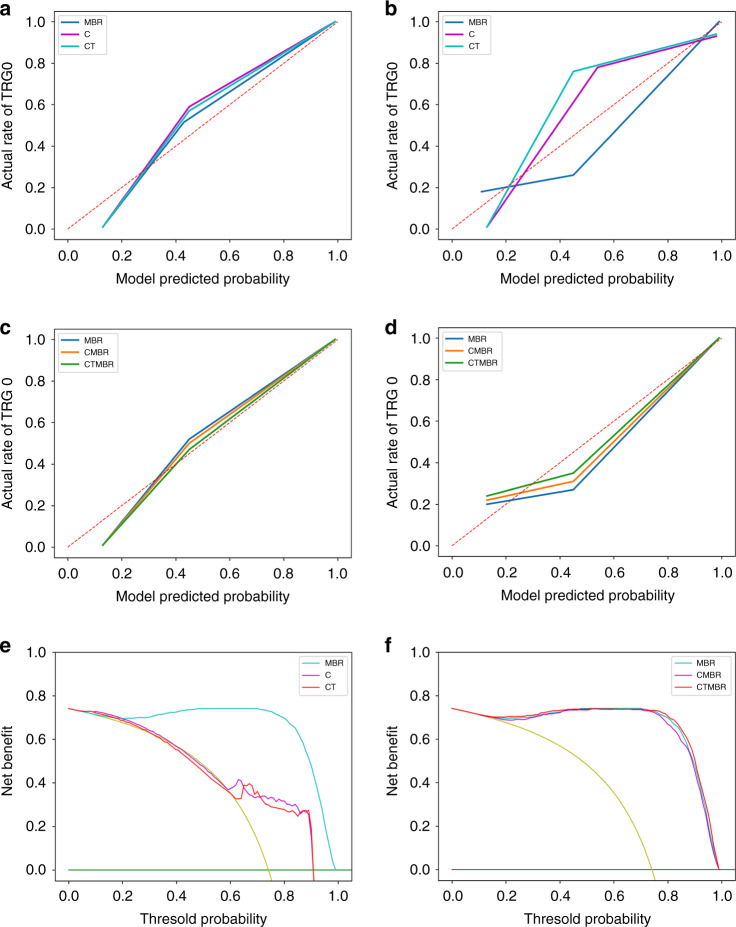
Calibration curves and decision curves for the five prediction models. Calibration curves for the non-imaging C, CT, and MBR models in the training (**a**) and validation (**b**) sets. Calibration curves for the radiomics models in the training (**c**) and validation (**d**) sets. The diagonal dashed red line represents the perfect performance of an ideal model. A closer fit to the diagonal dashed red line indicates a better prediction performance. Decision curve analysis for the C vs. CT vs. MBR models(**e**) and the MBR vs. CMBR vs. CTMBR models (**f**). The yellow line represents the assumption that all patients are TRG 0, whereas the green line represents the assumption that no patients are TRG 0.

The MBR signatures yielded mean AUCs of 0.9841 (95% CI, 0.9766–0.9914) in the training set and 0.7835 (95% CI, 0.6984–0.8686) in the validation set (Fig. 2c). The selected clinical baseline characteristics including pre-treatment CEA levels, MRI-T stage, and tumour histologic grade were integrated into the MBR signatures as independent clinical baseline risk characteristics and the CMBR model was generated. The selected radiomics features, including clinical baseline characteristics with their coefficients of the CMBR model, are listed in order from the highest to lowest in the Supplemental Material (eTables 3–7). The area under the ROC curve of the CMBR model achieved mean AUCs of 0.9891 (95% CI, 0.9854–0.9929) and 0.7871 (95% CI, 0.7057–0.8686) in the training and validation sets (Fig. 3d), respectively. The CTMBR model, which incorporated the MBR signatures, clinical baseline characteristics, and neoadjuvant treatment modalities, also demonstrated a satisfactory discrimination, with mean AUCs of 0.9907 (95% CI, 0.9869–0.9944) and 0.7916 (95% CI, 0.7570–0.8263) in the training and validation sets (Fig. 3e), respectively. The selected radiomics features including clinical baseline characteristics and neoadjuvant treatment modalities with their coefficients are listed in order from the highest to lowest in the Supplemental Material (eTables 8–12). The lower coefficient of clinical baseline characteristics and neoadjuvant treatment modalities indicated that their contributions to the models were lower than those of most of the radiomics features. In the above models, the MBR vs. CMBR model (*P* = 0.840), the MBR vs. CTMBR model (*P* = 0.890), and the CMBR vs. CTMBR model (*P* = 0.820) all performed similarly in the ROC analysis using the Delong test. The AUC, accuracy, PPV, and NPV of the five mentioned models are listed in Table 3.

**Table 3:** Performance of the C, CT, MBR, CMBR and CTMBR models.

Evaluation index	C model		CT model			MBR model		CMBR model		CTMBR model	
	Training set	Validation set	Training set	Validation set		Training set	Validation set	Training set	Validation set	Training set	Validation set
AUC	0.7053	0.6103	0.7512		0.6294	0.9841	0.7835	0.9891	0.7871	0.9907	0.7916
Accuracy	81.80%	66.42%	83.65%		70.11%	94.96%	92.59%	96.15%	93.19%	96.44%	93.33%
PPV	92.59%	86.02%	93.57%		88.48%	95.34%	93.45%	95.76%	93.65%	96.13%	94.02%
NPV	60.43%	40.23%	63.43%		44.37%	88.26%	89.74%	91.57%	91.03%	91.67%	91.08%

*AUC* area under the curve, *PPV* positive predictive value, *NPV* negative predictive value.

The calibration curves of the non-imaging C vs. CT models vs. MBR models (Fig. 3a, b) and MBR vs. CMBR vs. CTMBR models (Fig. 3c, d) demonstrated that the radiomics-based three prediction models showed good agreement between predicted and actual TRG 0 rates both in the training and validation sets. The decision curve analysis for the C vs. CT vs. MBR models and the MBR vs. CMBR vs. CTMBR models are presented in Fig. 3e, f. The decision curves showed that if the threshold probability ranged from 58% to 91% for the C model and 63% to 91% for the CT model, it could add more benefit (a net benefit could be derived) than a treat-all or treat-none scheme; if the threshold probability ranged from 13% to 99%, the radiomics models added more benefit than a treat-all or treat-none scheme as approximately more than 15% of patients underwent a CR [9].

## Discussion

Various factors seem to affect the pathologic response to neoadjuvant (chemo)radiotherapy in LARC. Clinical baseline characteristics including CEA levels, histologic and differentiation grade, and clinical stages showed close association with the degree of tumour regression [13, 30, 31]. Our data revealed that pre-treatment CEA levels, MRI-T stage, and tumour histologic grade were associated with CR, which was in accordance with the results of previous studies. Moreover, treatment modalities, such as concurrent chemoradiotherapy, the total radiation dose, and the total neoadjuvant therapy played an important role in this process [31–33]. However, there had been almost no consideration of treatment modalities in the prediction models of previous studies. Our real-world multicentre study explored the performance of the pre-treatment MBR signatures as well as the clinical baseline characteristics and neoadjuvant treatment modalities for tumour response prediction in patients with LARC. The performance of the radiomics based models were superior to the non-imaging models. Additionally, the performance of the clinical individualised models (CMBR and CTMBR) integrating the radiomics signatures, clinical baseline characteristics, and neoadjuvant treatment modalities could be improved to some extent, but it did not show significantly better discrimination than radiomics signatures alone. Meanwhile, features with the highest contribution to the model were mainly from MBR. Our results provided evidence that pre-treatment MBR signatures could be independent predictors of CR and seemed to reflect the true nature and extent of the tumour heterogeneity in LARC more accurately than clinical parameters.

Recently established MBR models for predicting tumour response were mainly from studies on relatively small or single-centre cohorts [20, 22, 34–36]. Because a wide variety of MR imagers exists not only among different facilities but also within the same institution, multicentre MRI-based and large-scale radiomics studies were previously difficult to conduct. With advancements in technology to minimise variability in acquisition parameters, multicentre radiomics has been attempted in rectal cancer [35, 37]. Liu et al. successfully developed and validated MBR signatures to predict distant metastasis within a multicentre and large-scale dataset [38]. The present study is in general accord with a previous study in that research based on high-quality MRI-based multicentre radiomics is practicable as we have shown a model with high accuracy in predicting treatment response using multicentre real-world data from a considerably large sample of patients.

According to our study, both the two non-imaging models had poor performance when compared with the radiomics signatures. Besides, when integrating radiomics signatures on the basis of non-imaging clinical models could significantly improve the performance of the model, which is similar to the results of other studies [39]. On the one hand, rectal tumours exhibit a remarkable heterogeneity that is significantly associated with disease stage and lymph node metastases [40, 41]. On the other hand, the genomic heterogeneity could translate to an intra-tumoural expression heterogeneity that could be assessed through radiomics [42]. Our results also indicated that the minor contribution degree of clinical baseline characteristics to the prediction model might be attributed to the correlation between radiomics and clinical baseline characteristics, which is in agreement with a previous study [36, 43]. In addition, radiomics signatures replaced the clinical macroscopic tumour details from multidimensions; thus, pre-treatment radiomics without clinical variables could achieve a good prediction of tumour shrinkage after neoadjuvant (chemo)radiotherapy in our study, which is consistent with the results of recent studies [34, 35].

More importantly, the discriminatory power of the prediction model integrating neoadjuvant treatment modalities could not be significantly improved. Neoadjuvant treatment modalities currently applied in the clinic are well known to have variable effects on CR. Addition of oxaliplatin to fluorouracil-based neoadjuvant chemoradiotherapy showed inconclusive results in terms of CR in clinical trials [33, 44]. Moreover, the recent intensification of 6 cycles chemotherapy using FOLFIRINOX before preoperative chemoradiotherapy significantly improved the pCR rate compared with preoperative chemoradiotherapy [45], and induction CAPEOX followed by chemoradiotherapy might not result in substantial tumour regression [46]. Furthermore, the chemotherapy cycles of consolidation therapy and radiotherapy modalities used were different in various studies, and the enrolled population was unequally targeted, perhaps resulting in the lack of consensus on the effect of CR in a large general population [47, 48]. Dose-escalated radiotherapy can also be associated with higher pCR rates, but this has not yet been confirmed in a randomised controlled trial [49]. The inconclusive results of the randomised clinical trials and recent meta-analysis could be attributed to factors including heterogeneous patient cohorts [50]. It suggested that intra-tumoural heterogeneity and histologic subtypes rather than conventional baseline characteristics, such as pre-treatment CEA, TNM staging, and tumour histologic type, were not well balanced between the experimental and control groups and thus might not be detected using the clinical method. Pre-treatment tumour intrinsic properties might have a greater impact on prognosis than neoadjuvant treatment modalities because intra-tumoural heterogeneity with distinct molecular and microenvironmental differences is more likely to foster treatment resistance and have poorer prognosis [51]. Therefore, determining the tumour intrinsic property and histological subtypes pre-treatment has great implications in choosing neoadjuvant treatment modalities to avoid overtreatment.

This study has several limitations. First, the collection of radiomics features was performed only on T2WI because the image features differed significantly between centres with more substantial variations in DWI [52]; T1-weighted (non-enhanced and contrast-enhanced) and dynamic contrast-enhanced sequences were not routinely recommended [53, 54]. We anticipated that the performance of our model might increase diagnostic accuracy with the inclusion of other standardised pre-processing pipeline MRI sequences. Second, no central review procedure was performed to determine reproducibility of the TRG classification. Third, since the variations in methodology concerning patient selection, image processing, outcome definition and statistics may have contributed to inconsistent findings between reports [55], the model also needs to be further optimised using better international standard engineering design with multilabel classification methods as well as further developed with more comprehensive integration of other molecular data, such as genomic and transcriptomic results. Finally, because the current treatment strategies are based on the TNM staging system, determining whether tumour response could significantly influence subsequent treatment strategies remains difficult.

We developed and validated multicentre prediction models based on pre-treatment MRI radiomics signatures, clinical baseline characteristics, and neoadjuvant treatment modalities to preliminarily screen CR to neoadjuvant (chemo)radiotherapy in patients with LARC. Pre-treatment MBR signatures seemed to reflect the true nature and extent of the tumour heterogeneity more accurately than clinical parameters, which could help identify LARC patients who can be offered organ preservation strategies and avoid overtreatment. The clinical usefulness of our radiomics model should be validated in larger, well-designed prospective multicentre studies in the future.

### Reporting summary

Further information on research design is available in the Nature Research Reporting Summary linked to this article.

## Supplementary information

Supplemental material

Reporting summary

## Data Availability

The data might be made available upon request, and some restrictions will apply.
