# Supplementary material for: MRI radiomics independent of clinical baseline characteristics and neoadjuvant treatment modalities predicts response to neoadjuvant therapy in rectal cancer
Source: Br J Cancer. 2022 Apr 2;127(2):249–57. doi: 10.1038/s41416-022-01786-7 (PMC9296479; doi:10.1038/s41416-022-01786-7)
Supplement: Supplementary file 1 — Supplemental material [file 41416_2022_1786_MOESM1_ESM.docx]

**Supplemental Material**

**CONTENT:**

**eAppendix I. Sample size calculation**

**eTable 1: Univariate and multivariate logistic regression model for the risk of TRG**

**eTable 2: Distribution of selected radiomics features in five training sets**

**eTable 3: Selected features and correlation coefficients of Training set 1 in CMBR model**

**eTable 4: Selected features and correlation coefficients of Training set 2 in CMBR model**

**eTable 5: Selected features and correlation coefficients of Training set 3 in CMBR model**

**eTable 6: Selected features and correlation coefficients of Training set 4 in CMBR model**

**eTable 7: Selected features and correlation coefficients of Training set 5 in CMBR model**

**eTable 8: Selected features and correlation coefficients of Training set 1 in CTMBR model**

**eTable 9: Selected features and correlation coefficients of Training set 2 in CTMBR model**

**eTable 10: Selected features and correlation coefficients of Training set 3 in CTMBR model**

**eTable 11: Selected features and correlation coefficients of Training set 4 in CTMBR model**

**eTable 12: Selected features and correlation coefficients of Training set 5 in CTMBR model**

**eAppendix I.** **Sample size calculation**

The predictive endpoint of this study was tumour regression grade (TRG), and patients were divided into TRG 0 and TGR 1-3 groups. It was reported in the literature that the rate of TRG 0 in rectal cancer was about 15-27%, therefore the ratio between the TGR 0 and TRG 1-3 groups was about 1:4. The sample size calculation used PASS 11.0 software. Set α value to 0.05, β value to 0.10, the ratio between groups was 1:4. We supposed that a diagnostic test must have an area under the receiver operating curve greater than 0.75. Previous literature used pre-treatment MRI radiomics for modelling with the area under the curve (AUC) achieving 0.90. The sample size required for the study is calculated to be at least 240 cases. Because the larger the sample size in machine learning, the better the stability of the model, 674 patients who met the requirements were finally included in this study.

| **eTable 1: Univariate and multivariate logistic regression model for the risk of TRG** | | | | |
| --- | --- | --- | --- | --- |
| **Variable** | **Univariate** | | **Multivariate** | |
|  | **OR (95% CI)** | ***P*** | **OR (95% CI)** | ***P*** |
| Sex(female vs. male) | 1.245(0.857-1.809) | .251 |  |  |
| Age (years) | 1.011(0.996-1.027) | .145 |  |  |
| Pre-treatment CEA(ng/ml)( ≥5 vs. <5) | 1.602(1.109-2.314) | .012 | 1.606(1.098-2.349) | .015 |
| Distance from anal verge(cm) |  |  |  |  |
| <5 | 1 |  |  |  |
| 5-10 | 1.155(0.815-1.635) | .418 |  |  |
| >10 | 1.512(0.314-7.267) | .606 |  |  |
| MRI-T Stage |  |  |  |  |
| 1-2 | 1 |  |  |  |
| 3 | 5.196(2.241-12.044) | .000 | 5.692(2.365-13.695) | <.001 |
| 4 | 6.989(2.805-17.409) | .000 | 9.217(3.519-24.140) | <.001 |
| Tumour histologic grade |  |  |  |  |
| Well differentiated adenocarcinoma | 1 |  |  |  |
| Moderately differentiated adenocarcinoma | 0.681(0.371-1.250) | .215 | 0.554(0.292-1.052) | .071 |
| Poorly differentiated adenocarcinoma | 0.369(0.167-0.819) | .014 | 0.255(0.109-0.596) | .002 |
| Signet ring cell cancer or mucinous adenocarcinoma | 1.552(0.173-13.890) | .694 | 1.302(0.143-11.838) | .815 |
| Uncertain differentiation type | 1.211(0.583-2.515) | .608 | 1.120(0.512-2.449) | .776 |
| Time interval between neoadjuvant (chemo)radiotherapy and surgery (weeks) | 1.002(0.998-1.007) | .345 |  |  |
| Abbreviations: TRG, tumour regression grade; OR, odds ratio; CI, confidence interval; CEA, carcinoembryonic antigen; MRI-T stage, MRI-predicted T stage; | | | | |

| **eTable 2: Distribution of selected radiomics features in five training sets** | | | | | |
| --- | --- | --- | --- | --- | --- |
|  | **Number of selected features** | | | | |
| **Features** | **Training set 1** | **Training set 2** | **Training set 3** | **Training set 4** | **Training set 5** |
| Histogram of oriented gradient features(HOG) | 16 | 16 | 16 | 13 | 16 |
| Texture features | 1 | 3 | 2 | 2 | 0 |
| Wavelet features | 3 | 2 | 3 | 4 | 4 |
| Statistical features | 10 | 9 | 9 | 11 | 10 |

**eTable 3: Selected features and correlation coefficients of Training set 1 in CMBR model**

| **Selected Features in Set 1** | **Correlation Coefficient** |
| --- | --- |
| hog_2_1_2_1_3D_Histogram | 0.096201940 |
| hog_0_0_1_11_3D_Histogram | 0.067302671 |
| hog_0_0_2_18_3D_Histogram | 0.066305705 |
| hog_1_0_0_15_3D_Histogram | 0.058143129 |
| hog_1_0_0_8_3D_Histogram | 0.056632466 |
| hog_1_0_1_1_3D_Histogram | 0.055641847 |
| hog_1_0_2_16_3D_Histogram | 0.043648184 |
| hog_2_0_0_17_3D_Histogram | 0.041300113 |
| hog_1_1_0_5_3D_Histogram | 0.040755997 |
| hog_1_2_1_11_3D_Histogram | 0.038004239 |
| hog_2_0_0_3_3D_Histogram | 0.034051901 |
| hog_2_1_0_9_3D_Histogram | 0.032258516 |
| statistic_0_1_-1-Homogeneity | 0.030341469 |
| hog_2_1_0_11_3D_Histogram | 0.030006568 |
| hog_2_0_1_15_3D_Histogram | 0.029642615 |
| hog_2_1_0_10_3D_Histogram | 0.028613730 |
| hog_2_1_0_17_3D_Histogram | 0.026659556 |
| statistic_0_1_-1-Entropy | 0.025346664 |
| statistic_1_0_-1-Homogeneity | 0.022124036 |
| statistic_-1_0_-1-MaxProbability | 0.020618622 |
| statistic_-1_0_-1-lnverse_Variance | 0.019941987 |
| wavelet_LLH-Histogram-Variance | 0.019000000 |
| statistic_-1_-1_0-SumMean | 0.016494946 |
| statistic_-1_1_0-Variance | 0.015300057 |
| statistic_1_-1_-1-Entropy | 0.015104907 |
| statistic_-1_1_0-Homogeneity | 0.014985744 |
| statistic_-1_-1_-1-ClusterTendency | 0.014004369 |
| wavelet_HHL-Histogram-Entropy | 0.009082543 |
| MRI-T stage | 0.008960032 |
| Wavelet-LHH - Histogram - Energy | 0.008830211 |
| texture_(2)Histogram-Variance | 0.008634299 |
| Pre-treatment CEA | 0.003060937 |
| Tumour histologic type | 0.003000000 |

Abbreviations: CEA carcinoembryonic antigen, MRI-T stage MRI-predicted T stage

**eTable 4: Selected features and correlation coefficients of Training set 2 in CMBR model**

| **Selected Features in Set 2** | **Correlation Coefficient** |
| --- | --- |
| texture_(15)GTSDM-Entropy | 0.090955933 |
| statistic_-1_0_-1-Inertia | 0.077884756 |
| statistic_1_0_-1-ClusterTendency | 0.063329379 |
| statistic_1_-1_-1-Inertia | 0.061261376 |
| Texture-Histogram-Kurtosis | 0.054788892 |
| hog_0_2_2_6_3D_Histogram | 0.049885439 |
| statistic_-1_-1_0-Contrast | 0.049011888 |
| wavelet_HLH-Histogram-Mean | 0.046293545 |
| hog_0_0_0_7_3D_Histogram | 0.039456221 |
| hog_2_1_1_17_3D_Histogram | 0.037789256 |
| hog_1_1_1_10_3D_Histogram | 0.035693299 |
| statistic_-1_-1_-1-Homogeneity | 0.032003121 |
| hog_1_0_0_15_3D_Histogram | 0.031783308 |
| hog_1_0_0_8_3D_Histogram | 0.030678636 |
| statistic_0_-1_-1-ClusterTendency | 0.028191587 |
| statistic_1_-1_-1-Homogeneity | 0.024421958 |
| hog_2_0_1_4_3D_Histogram | 0.024323598 |
| hog_0_2_0_14_3D_Histogram | 0.023532071 |
| statistic_1_1_-1-Max Probability | 0.023316986 |
| hog_0_1_0_16_3D_Histogram | 0.022068050 |
| hog_2_0_0_1_3D_Histogram | 0.020858967 |
| statistic_-1_1_-1-MaxProbability | 0.019333390 |
| wavelet_LLL-Histogram-Variance | 0.019123275 |
| Texture-Histogram-Skewness | 0.018925671 |
| hog_2_2_2_11_3D_Histogram | 0.018412430 |
| hog_1_0_1_1_3D_Histogram | 0.017955389 |
| hog_0_2_1_18_3D_Histogram | 0.015035142 |
| hog_2_0_0_17_3D_Histogram | 0.014581740 |
| hog_2_0_1_6_3D_Histogram | 0.006692400 |
| hog_2_1_0_6_3D_Histogram | 0.001185468 |
| MRI-T stage | 0.000875120 |
| Pre-treatment CEA | 0.000223009 |
| Tumour histologic type | 0.000128700 |

Abbreviations: CEA carcinoembryonic antigen, MRI-T stage MRI-predicted T stage

**eTable 5: Selected features and correlation coefficients of Training set 3 in CMBR model**

| **Selected Features in Set 3** | **Correlation Coefficient** |
| --- | --- |
| statistic_-1_-1_-1-ClusterShade | 0.094201940 |
| wavelet_HHH-Histogram-Entropy | 0.083302671 |
| statistic_0_1_0-Inertia | 0.077143129 |
| hog_0_0_1_16_3D_Histogram | 0.057305705 |
| hog_2_2_2_16_3D_Histogram | 0.045641847 |
| hog_0_1_1_11_3D_Histogram | 0.045632466 |
| statistic_0_-1_-1-Contrast | 0.045341469 |
| hog_2_0_0_16_3D_Histogram | 0.043300113 |
| hog_1_2_0_19_3D_Histogram | 0.040755997 |
| hog_0_0_0_3_3D_Histogram | 0.038006568 |
| hog_2_2_1_11_3D_Histogram | 0.037648184 |
| hog_2_0_2_7_3D_Histogram | 0.032494946 |
| hog_1_1_2_5_3D_Histogram | 0.032346664 |
| texture_(33)GLZSM-LargeZoneSizeEmphasis | 0.031204239 |
| wavelet_HHL-Histogram-Energy | 0.029258516 |
| hog_1_2_2_10_3D_Histogram | 0.027642615 |
| statistic_1_-1_-1-Sum Mean | 0.025941987 |
| statistic_1_1_-1-Inverse Variance | 0.021634299 |
| statistic_1_1_-1-Inertia | 0.021104907 |
| statistic_0_-1_-1-Variance | 0.020959556 |
| hog_0_2_2_14_3D_Histogram | 0.020051901 |
| hog_0_1_2_6_3D_Histogram | 0.018004369 |
| hog_0_2_1_0_3D_Histogram | 0.017124036 |
| hog_1_1_2_8_3D_Histogram | 0.016618622 |
| hog_2_2_0_10_3D_Histogram | 0.015613731 |
| statistic_0_1_-1-Variance | 0.015300057 |
| hog_0_2_0_3_3D_Histogram | 0.009960032 |
| wavelet_LHL-Histogram-Variance | 0.009082543 |
| texture_(38)GLZSM-LargeZone/LowGrayEmphasis | 0.007830211 |
| MRI-T stage | 0.007800758 |
| statistic_1_0_-1-Contrast | 0.005985744 |
| Pre-treatment CEA | 0.004439063 |
| Tumour histologic type | 0.001321115 |

Abbreviations: CEA carcinoembryonic antigen, MRI-T stage MRI-predicted T stage

**eTable 6: Selected features and correlation coefficients of Training set 4 in CMBR model**

| **Selected Features in Set 4** | **Correlation Coefficient** |
| --- | --- |
| statistic_-1_0_0-Contrast | 0.100533539 |
| wavelet_LLL-Histogram-Skewness | 0.096682919 |
| statistic_0_1_0-Contrast | 0.053271210 |
| hog_0_0_1_19_3D_Histogram | 0.052757359 |
| wavelet_LLL-Histogram-Kurtosis | 0.049343969 |
| hog_0_1_1_12_3D_Histogram | 0.046379643 |
| statistic_-1_-1_0-Homogeneity | 0.039916968 |
| hog_2_0_2_6_3D_Histogram | 0.039487711 |
| hog_1_2_2_11_3D_Histogram | 0.038487711 |
| statistic_0_1_0-MaxProbability | 0.038422045 |
| texture_(33)GLZSM-LargeZoneSizeEmphasis | 0.036176586 |
| statistic_1_0_-1-Homogeneity | 0.034881395 |
| hog_1_2_2_10_3D_Histogram | 0.033514326 |
| texture_(38)GLZSM-LargeZone/LowGrayEmphasis | 0.032043691 |
| hog_0_0_1_7_3D_Histogram | 0.030175114 |
| hog_1_1_1_13_3D_Histogram | 0.030105598 |
| statistic_-1_1_1_Variance | 0.028805561 |
| wavelet_HLL-Histogram-Entropy | 0.025752516 |
| Wavelet-HLL - Histogram - Kurtosis | 0.024104088 |
| hog_2_2_0_12_3D_Histogram | 0.021029769 |
| statistic_-1_0_0-Inertia | 0.020837107 |
| hog_1_0_0_8_3D_Histogram | 0.019571911 |
| statistic_-1_-1_-1-Homogeneity | 0.018753509 |
| hog_2_2_0_17_3D_Histogram | 0.017534178 |
| hog_0_2_0_6_3D_Histogram | 0.015684539 |
| hog_0_1_2_14_3D_Histogram | 0.013306027 |
| hog_2_0_1_16_3D_Histogram | 0.010250777 |
| statistic_1_-1_-1-ClusterShade | 0.007711224 |
| statistic_1_1_-1_SumMean | 0.007318194 |
| Pre-treatment CEA | 0.006696372 |
| MRI-T stage | 0.004477484 |
| Tumour histologic type | 0.004248920 |
| statistic_1_1_-1-ClusterShade | 0.001738040 |

Abbreviations: CEA carcinoembryonic antigen, MRI-T stage MRI-predicted T stage

**eTable 7: Selected features and correlation coefficients of Training set 5 in CMBR model**

| **Selected Features in Set 5** | **Correlation Coefficient** |
| --- | --- |
| statistic_1_1_-1-Homogeneity | 0.120142246 |
| hog_2_0_2_9_3D_Histogram | 0.080553002 |
| statistic_0_0_-1-Homogeneity | 0.057652657 |
| statistic_1_-1_-1-Entropy | 0.056329314 |
| wavelet_HLH-Histogram-Mean | 0.052257258 |
| statistic_-1_0_-1-Homogeneity | 0.046979313 |
| statistic_-1_-1_0-Contrast | 0.045768014 |
| statistic_0_1_0-ClusterTendency | 0.039574390 |
| wavelet_LHL-Histogram-Kurtosis | 0.035783428 |
| hog_2_0_1_4_3D_Histogram | 0.034526103 |
| wavelet_HHL-Histogram-Variance | 0.028026909 |
| statistic_-1_0_-1-Inertia | 0.027329236 |
| hog_1_0_2_18_3D_Histogram | 0.027145557 |
| hog_2_2_1_5_3D_Histogram | 0.026722797 |
| Wavelet-HLL - Histogram - Kurtosis | 0.025730513 |
| hog_0_1_0_3_3D_Histogram | 0.024936050 |
| statistic_-1_1_-1-Homogeneity | 0.024117429 |
| hog_0_0_1_6_3D_Histogram | 0.022562137 |
| hog_2_1_0_6_3D_Histogram | 0.021366487 |
| hog_0_2_0_12_3D_Histogram | 0.020947570 |
| hog_0_0_1_7_3D_Histogram | 0.020659429 |
| statistic_-1_-1_0-ClusterShade | 0.020345980 |
| hog_2_0_0_1_3D_Histogram | 0.020342387 |
| hog_1_2_0_16_3D_Histogram | 0.016210293 |
| hog_0_1_2_13_3D_Histogram | 0.015801498 |
| hog_0_2_2_6_3D_Histogram | 0.015251549 |
| hog_1_1_1_17_3D_Histogram | 0.015082543 |
| hog_0_0_2_4_3D_Histogram | 0.014893159 |
| statistic_-1_1_0-Homogeneity | 0.014751133 |
| hog_0_1_1_11_3D_Histogram | 0.014116827 |
| MRI-T stage | 0.009920690 |
| Pre-treatment CEA | 0.003174102 |
| Tumour histologic type | 0.001000000 |

Abbreviations: CEA carcinoembryonic antigen, MRI-T stage MRI-predicted T stage

**eTable 8: Selected features and correlation coefficients of Training set 1 in CTMBR model**

| **Selected Features in Set 1** | **Correlation Coefficient** |
| --- | --- |
| hog_0_0_1_11_3D_Histogram | 0.068302671 |
| hog_0_0_2_18_3D_Histogram | 0.067305705 |
| hog_2_1_2_1_3D_Histogram | 0.062201940 |
| hog_1_0_0_15_3D_Histogram | 0.059143129 |
| hog_1_0_0_8_3D_Histogram | 0.057632466 |
| hog_1_0_1_1_3D_Histogram | 0.056641847 |
| hog_1_0_2_16_3D_Histogram | 0.044648184 |
| hog_2_0_0_17_3D_Histogram | 0.042300113 |
| hog_1_1_0_5_3D_Histogram | 0.041755997 |
| hog_1_2_1_11_3D_Histogram | 0.039004239 |
| hog_2_0_0_3_3D_Histogram | 0.035051901 |
| hog_2_1_0_9_3D_Histogram | 0.033258516 |
| statistic_0_1_-1-Homogeneity | 0.031341469 |
| hog_2_1_0_11_3D_Histogram | 0.031006568 |
| hog_2_0_1_15_3D_Histogram | 0.030642615 |
| hog_2_1_0_10_3D_Histogram | 0.029613730 |
| hog_2_1_0_17_3D_Histogram | 0.027659556 |
| statistic_0_1_-1-Entropy | 0.026346664 |
| statistic_1_0_-1-Homogeneity | 0.023124036 |
| statistic_-1_0_-1-MaxProbability | 0.021618622 |
| statistic_-1_0_-1-lnverse_Variance | 0.020941987 |
| wavelet_LLH-Histogram-Variance | 0.020000000 |
| statistic_-1_-1_0-SumMean | 0.017494946 |
| statistic_-1_1_0-Variance | 0.016300057 |
| statistic_1_-1_-1-Entropy | 0.016104907 |
| statistic_-1_1_0-Homogeneity | 0.015985744 |
| statistic_-1_-1_-1-ClusterTendency | 0.015004369 |
| wavelet_HHL-Histogram-Entropy | 0.010082543 |
| MRI-T stage | 0.009960032 |
| Wavelet-LHH - Histogram - Energy | 0.009830211 |
| texture_(2)Histogram-Variance | 0.009634299 |
| Pre-treatment CEA | 0.004060937 |
| Tumour histologic type | 0.004000000 |
| Radiation modality | 0.000891301 |
| Consolidation chemotherapy | 0.000568137 |
| Induction chemotherapy | 0.000321437 |
| Concurrent chemotherapy | 0.000219125 |

Abbreviations: CEA carcinoembryonic antigen, MRI-T stage MRI-predicted T stage

**eTable 9: Selected features and correlation coefficients of Training set 2 in CTMBR model**

| **Selected Features in Set 2** | **Correlation Coefficient** |
| --- | --- |
| texture_(15)GTSDM-Entropy | 0.089955933 |
| statistic_-1_0_-1-Inertia | 0.075884756 |
| statistic_1_0_-1-ClusterTendency | 0.060329379 |
| statistic_1_-1_-1-Inertia | 0.057261376 |
| Texture-Histogram-Kurtosis | 0.049788892 |
| statistic_-1_-1_0-Contrast | 0.048011888 |
| hog_0_2_2_6_3D_Histogram | 0.045885439 |
| wavelet_HLH-Histogram-Mean | 0.043293545 |
| hog_2_1_1_17_3D_Histogram | 0.036789256 |
| hog_0_0_0_7_3D_Histogram | 0.035456221 |
| hog_1_1_1_10_3D_Histogram | 0.034693299 |
| hog_1_0_0_8_3D_Histogram | 0.032678636 |
| statistic_-1_-1_-1-Homogeneity | 0.030003121 |
| hog_1_0_0_15_3D_Histogram | 0.028783308 |
| hog_2_0_1_4_3D_Histogram | 0.028323598 |
| hog_0_2_0_14_3D_Histogram | 0.026532071 |
| statistic_0_-1_-1-ClusterTendency | 0.026191587 |
| statistic_1_-1_-1-Homogeneity | 0.025421958 |
| hog_0_1_0_16_3D_Histogram | 0.025068050 |
| statistic_-1_1_-1-MaxProbability | 0.023333390 |
| hog_0_2_1_18_3D_Histogram | 0.023235142 |
| Texture-Histogram-Skewness | 0.021925671 |
| hog_2_0_0_1_3D_Histogram | 0.021858967 |
| statistic_1_1_-1-Max Probability | 0.021316986 |
| wavelet_LLL-Histogram-Variance | 0.021123275 |
| hog_2_2_2_11_3D_Histogram | 0.019412430 |
| hog_1_0_1_1_3D_Histogram | 0.018955389 |
| hog_2_0_0_17_3D_Histogram | 0.016581740 |
| hog_2_0_1_6_3D_Histogram | 0.005692400 |
| hog_2_1_0_6_3D_Histogram | 0.002185468 |
| Tumour histologic type | 0.001871300 |
| Pre-treatment CEA | 0.000776991 |
| Concurrent chemotherapy | 0.000468013 |
| Consolidation chemotherapy | 0.000458290 |
| Induction chemotherapy | 0.000203231 |
| MRI-T stage | 0.000124880 |
| Radiation modality | 0.000124124 |

Abbreviations: CEA carcinoembryonic antigen, MRI-T stage MRI-predicted T stage

**eTable 10: Selected features and correlation coefficients of Training set 3 in CTMBR model**

| **Selected Features in Set 3** | **Correlation Coefficient** |
| --- | --- |
| statistic_-1_-1_-1-ClusterShade | 0.095201940 |
| wavelet_HHH-Histogram-Entropy | 0.082302671 |
| statistic_0_1_0-Inertia | 0.077143129 |
| hog_0_0_1_16_3D_Histogram | 0.055305705 |
| hog_2_2_2_16_3D_Histogram | 0.044641847 |
| statistic_0_-1_-1-Contrast | 0.042341469 |
| hog_0_1_1_11_3D_Histogram | 0.040632466 |
| hog_2_0_0_16_3D_Histogram | 0.040300113 |
| hog_1_2_0_19_3D_Histogram | 0.039755997 |
| hog_0_0_0_3_3D_Histogram | 0.036006568 |
| hog_2_2_1_11_3D_Histogram | 0.033648184 |
| hog_1_1_2_5_3D_Histogram | 0.033346664 |
| hog_1_2_2_10_3D_Histogram | 0.030642615 |
| hog_2_0_2_7_3D_Histogram | 0.030494946 |
| texture_(33)GLZSM-LargeZoneSizeEmphasis | 0.030204239 |
| wavelet_HHL-Histogram-Energy | 0.027258516 |
| statistic_1_1_-1-Inverse Variance | 0.025634299 |
| statistic_1_1_-1-Inertia | 0.023104907 |
| statistic_0_-1_-1-Variance | 0.022959556 |
| statistic_1_-1_-1-Sum Mean | 0.021941987 |
| hog_0_2_1_0_3D_Histogram | 0.021124036 |
| hog_0_2_2_14_3D_Histogram | 0.021051901 |
| hog_1_1_2_8_3D_Histogram | 0.019618622 |
| hog_0_1_2_6_3D_Histogram | 0.019004369 |
| statistic_0_1_-1-Variance | 0.018300057 |
| hog_2_2_0_10_3D_Histogram | 0.017613731 |
| texture_(38)GLZSM-LargeZone/LowGrayEmphasis | 0.012830211 |
| wavelet_LHL-Histogram-Variance | 0.010082543 |
| hog_0_2_0_3_3D_Histogram | 0.008960032 |
| MRI-T stage | 0.007500758 |
| statistic_1_0_-1-Contrast | 0.006985744 |
| Pre-treatment CEA | 0.002439063 |
| Induction chemotherapy | 0.000437592 |
| Consolidation chemotherapy | 0.000356023 |
| Tumour histologic type | 0.000321115 |
| Concurrent chemotherapy | 0.000269101 |
| Radiation modality | 0.000237285 |

Abbreviations: CEA carcinoembryonic antigen, MRI-T stage MRI-predicted T stage

**eTable 11: Selected features and correlation coefficients of Training set 4 in CTMBR model**

| **Selected Features in Set 4** | **Correlation Coefficient** |
| --- | --- |
| statistic_-1_0_0-Contrast | 0.099533539 |
| wavelet_LLL-Histogram-Skewness | 0.085282919 |
| statistic_0_1_0-Contrast | 0.055271210 |
| hog_0_0_1_19_3D_Histogram | 0.053757359 |
| hog_0_1_1_12_3D_Histogram | 0.049379643 |
| hog_2_0_2_6_3D_Histogram | 0.043487711 |
| wavelet_LLL-Histogram-Kurtosis | 0.041343969 |
| hog_1_2_2_11_3D_Histogram | 0.040487711 |
| statistic_0_1_0-MaxProbability | 0.040422045 |
| hog_1_2_2_10_3D_Histogram | 0.036514326 |
| statistic_-1_-1_0-Homogeneity | 0.035916968 |
| texture_(33)GLZSM-LargeZoneSizeEmphasis | 0.033176586 |
| hog_1_1_1_13_3D_Histogram | 0.033105598 |
| hog_0_0_1_7_3D_Histogram | 0.032175114 |
| statistic_1_0_-1-Homogeneity | 0.031881395 |
| texture_(38)GLZSM-LargeZone/LowGrayEmphasis | 0.030043691 |
| statistic_-1_1_1_Variance | 0.026805561 |
| hog_2_2_0_12_3D_Histogram | 0.025029769 |
| wavelet_HLL-Histogram-Entropy | 0.024352516 |
| Wavelet-HLL - Histogram - Kurtosis | 0.024104088 |
| hog_0_2_0_6_3D_Histogram | 0.020684539 |
| hog_1_0_0_8_3D_Histogram | 0.020571911 |
| statistic_-1_0_0-Inertia | 0.018837107 |
| hog_2_2_0_17_3D_Histogram | 0.018534178 |
| statistic_-1_-1_-1-Homogeneity | 0.017753509 |
| hog_0_1_2_14_3D_Histogram | 0.017306027 |
| hog_2_0_1_16_3D_Histogram | 0.011250777 |
| Pre-treatment CEA | 0.007696372 |
| MRI-T stage | 0.006477484 |
| statistic_1_1_-1_SumMean | 0.006318194 |
| Tumour histologic type | 0.005248920 |
| statistic_1_-1_-1-ClusterShade | 0.003711224 |
| statistic_1_1_-1-ClusterShade | 0.001261960 |
| Consolidation chemotherapy | 0.000857292 |
| Radiation modality | 0.000840283 |
| Concurrent chemotherapy | 0.000394756 |
| Induction chemotherapy | 0.000183749 |

Abbreviations: CEA carcinoembryonic antigen, MRI-T stage MRI-predicted T stage

**eTable 12: Selected features and correlation coefficients of Training set 5 in CTMBR model**

| **Selected Features in Set 5** | **Correlation Coefficient** |
| --- | --- |
| statistic_1_1_-1-Homogeneity | 0.117142246 |
| hog_2_0_2_9_3D_Histogram | 0.082553002 |
| statistic_0_0_-1-Homogeneity | 0.055652657 |
| wavelet_HLH-Histogram-Mean | 0.047257258 |
| statistic_1_-1_-1-Entropy | 0.045329314 |
| statistic_-1_0_-1-Homogeneity | 0.042979313 |
| statistic_-1_-1_0-Contrast | 0.042768014 |
| statistic_0_1_0-ClusterTendency | 0.036574390 |
| hog_2_0_1_4_3D_Histogram | 0.035526103 |
| wavelet_LHL-Histogram-Kurtosis | 0.034783428 |
| hog_1_0_2_18_3D_Histogram | 0.029145557 |
| hog_0_1_0_3_3D_Histogram | 0.028936050 |
| wavelet_HHL-Histogram-Variance | 0.027026909 |
| Wavelet-HLL - Histogram - Kurtosis | 0.026730513 |
| statistic_-1_0_-1-Inertia | 0.026329236 |
| hog_2_2_1_5_3D_Histogram | 0.025722797 |
| hog_2_0_0_1_3D_Histogram | 0.024342387 |
| hog_0_2_0_12_3D_Histogram | 0.023947570 |
| hog_0_0_1_6_3D_Histogram | 0.023562137 |
| hog_2_1_0_6_3D_Histogram | 0.023366487 |
| hog_0_0_1_7_3D_Histogram | 0.022659429 |
| statistic_-1_1_-1-Homogeneity | 0.022117429 |
| hog_1_2_0_16_3D_Histogram | 0.020210293 |
| hog_0_1_1_11_3D_Histogram | 0.019116827 |
| statistic_-1_-1_0-ClusterShade | 0.018345980 |
| hog_0_2_2_6_3D_Histogram | 0.018251549 |
| hog_0_0_2_4_3D_Histogram | 0.017893159 |
| hog_0_1_2_13_3D_Histogram | 0.016801498 |
| hog_1_1_1_17_3D_Histogram | 0.016082543 |
| MRI-T stage | 0.011920690 |
| statistic_-1_1_0-Homogeneity | 0.010751133 |
| Pre-treatment CEA | 0.002774102 |
| Tumour histologic type | 0.002000000 |
| Induction chemotherapy | 0.000476936 |
| Consolidation chemotherapy | 0.000427855 |
| Radiation modality | 0.000384981 |
| Concurrent chemotherapy | 0.000110228 |

Abbreviations: CEA carcinoembryonic antigen, MRI-T stage MRI-predicted T stage
